# Lung Adenocarcinoma with Metachronous Ovarian Metastasis: a long survival case report

**DOI:** 10.1186/s12905-021-01284-7

**Published:** 2021-04-14

**Authors:** Shuyang Yao, Leiming Wang, Xiaoru Tian, Yi Zhang

**Affiliations:** 1 Department of Thoracic Surgery, Xuanwu Hospital, The Capital Medical University, Beijing, China; 2 Department of Pathology, Xuanwu Hospital, The Capital Medical University, Beijing, China

**Keywords:** Lung adenocarcinoma, Ovarian metastasis, Debulking surgery, Targeted therapy, Case report

## Abstract

**Background:**

Lung adenocarcinoma which invades ovaries is very rare. However, with the increase of long-survival female lung cancer, more patients will suffer ovarian metastasis. On grounds of the paucity of reported cases, the clinicopathological features and treatment strategies remain unknown.

**Case presentation:**

This patient was stage IV lung adenocarcinoma at first diagnosis. Following multiple-line systemic treatments, she experienced extensive pelvic metastasis. After debulking surgery and reevaluation about the drive genes, she was administered by targeted therapy. Up to now, the patient has shown no evidence of progression for 8 years after the initial diagnosis of primary lung cancer and 46 months after her ovarian metastasis.

**Conclusion:**

The comprehensive treatment modality for the bilateral ovarian metastasis is effective in clinical course.

## Introduction

Compared with common metastases like bone, brain, liver, adrenal glands and so on, lung cancer metastasis to uncommon sites occurs in less than 5% of cases, especially associated with a worse outcome, and the incidence of metastatic adenocarcinoma to the ovaries is only 0.07% [1]. This frequency, however, is increasing due to the rising incidence of lung adenocarcinoma in women and the prolonged survival time of lung cancer. Because of the paucity of reported cases [2, 3], the clinicopathological features and treatment strategies of these tumors remain unknown. Here, we report a long surviving patient with ovarian metastasis from lung adenocarcinoma.

## Case presentation

A 60-year-old woman presented with cough and dyspnea in March 2012. Computed tomography (CT) scans showed a left lower lobe mass, as well as plural metastasis and pleural effusion. A pathological diagnosis of adenocarcinoma was performed by pleural biopsy. Lung tumor tissue was wild-type of epidermal growth factor receptor (EGFR) by the method of real-time polymerase chain reaction. Then she received two-line chemotherapies (docetaxel (75 mg/m^2^) + carboplatin (4 area under the curve) × 6 cycles and pemetrexed (500 mg/m^2^) + cisplatin (60 mg/m^2^) × 6 cycles, respectively) with the best response of stable disease (SD) for each regimen, but it finally failed for progressive disease (PD) with new metastasis in bones and brain. Gefitinib had been administrated as her third-line treatment since July 2014.

In June 2016, she was admitted with the complaint of abdominal discomfort and obvious weight loss. CT scans of the ovarian tumor revealed the admixture of enhanced solid components and cysts, left 4. 6 × 3. 5 cm, right 2. 6 × 2. 9 cm, with extensive peritoneal thickening and massive ascites. Then, she underwent tumor debulking surgery. The tumor invaded not only bilateral ovaries, but also peritoneum, bladder, uterine. Even a great many of small nodules scattered on the surface of intestinal tubes. The following procedures were done: bilateral salpingooophorectomy, partial omentectomy, and peritoneal washings with cisplatin 80 mg.

**Fig. 1:**
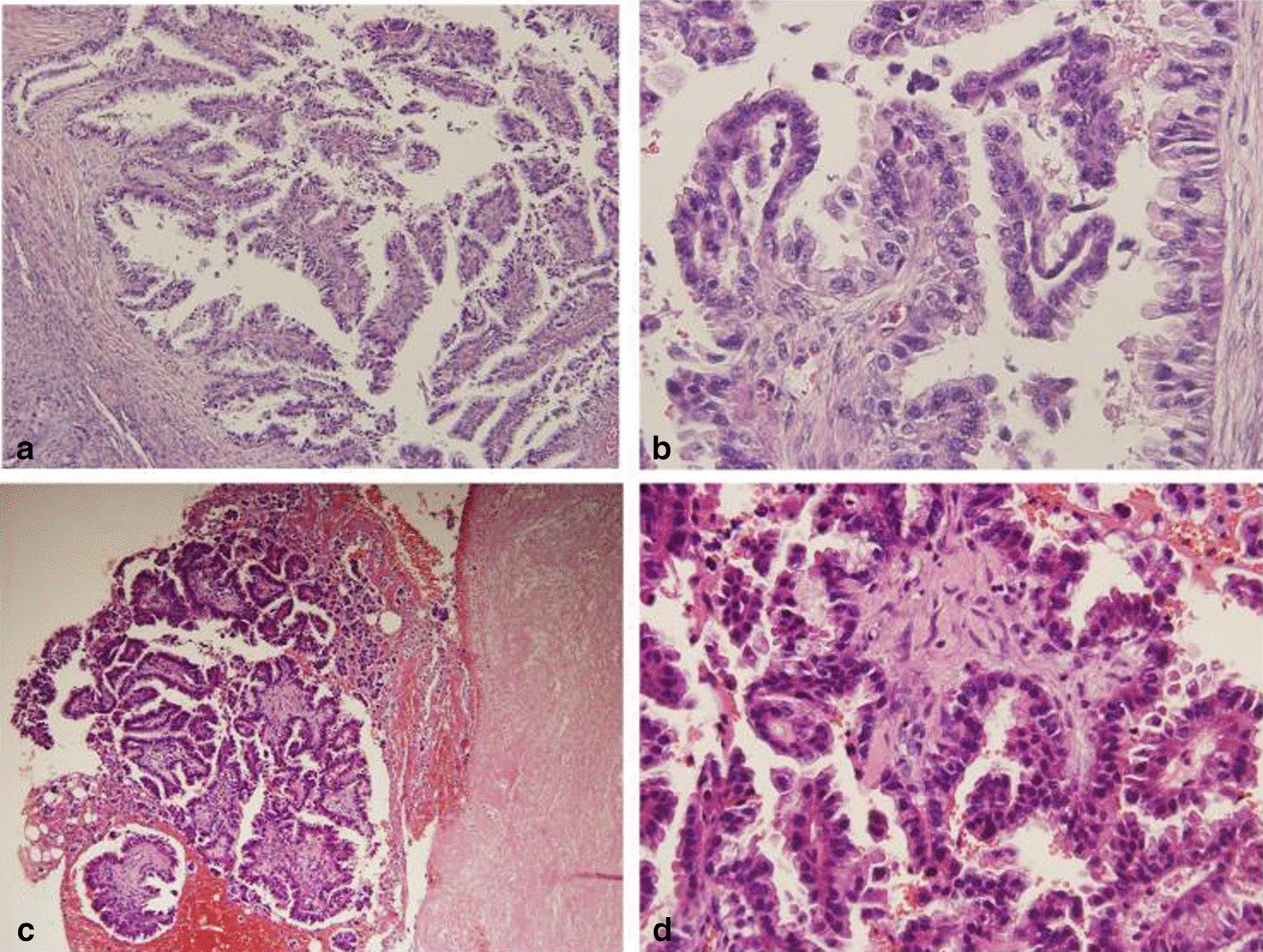
In 2006, from debulking surgery, tumor cells are arranged into glandular tubular and papillary structures with apical snouts and huge, hyperchromatic nuclei. **a** H&E, ×100; **b** H&E, ×400; In 2002, from pleural biopsy, tumor cells are arranged into glandular tubular and papillary structures with apical snouts and huge, hyperchromatic nuclei. **a** H&E, ×100; **b** H&E, ×400

**Fig. 2:**
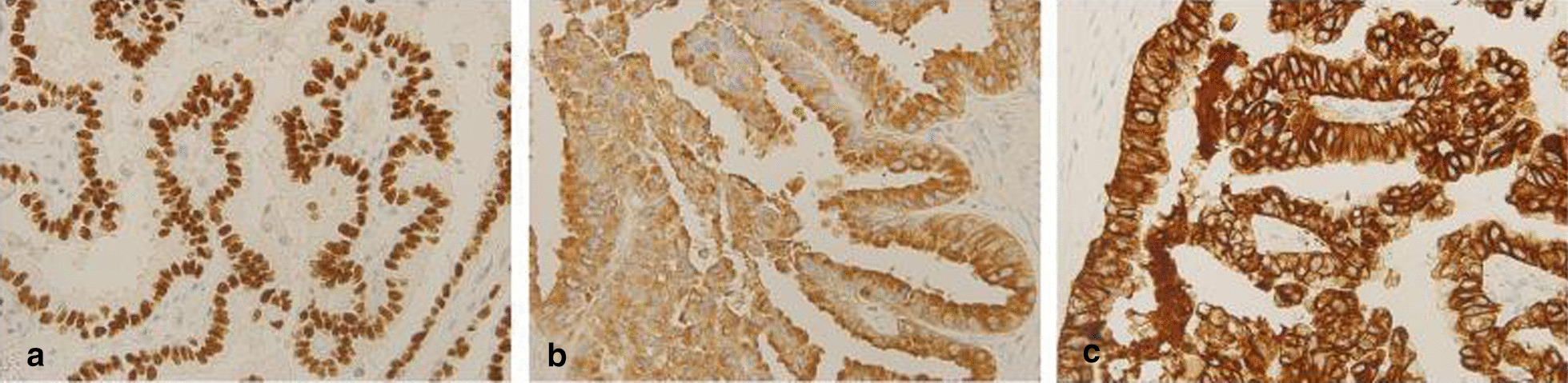
In tissue from the ovarian debulking surgery, **a** tumor cells are immunopositive for TTF1. IHC, ×400; **b** Tumor cells are immunopositive for NapsinA. IHC, ×400; **c** Tumor cells are immunopositive for CK7. IHC, ×400

Pathology showed a moderately differentiated papillary adenocarcinoma in ovarian. The tumor cells were arranged into glandular tubular and papillary structures with apical snouts and huge, hyperchromatic nuclei (Fig. 1a, b). Mucous epithelium could be seen in some areas with stromal fibrous proliferation. Tumor cells were immunopositive for thyroid transcription factor 1 (TTF-1), Napsin-A and cytokeratin (CK)-7 (Fig. 2a–c), and negative for paired box 8 (PAX-8), estrogen receptors (ER), progesterone receptor (PR) and CK20. Combined with the initial results of pleural biopsy (Fig. 1c, d), the diagnosis of ovarian metastasis from lung adenocarcinoma was established.

Evaluated by the testing method as before, it showed EGFR T790M mutation in exon 20. Then the patient received oral administration of osimertinib (80 mg once a day). Up to now, the patient has shown no evidence of progression on regular follow-up for 8 years after the initial diagnosis of primary lung cancer and 46 months after her ovarian metastasis.

## Discussion and conclusions

Metastatic ovarian tumors account for 4.2% of malignant ovarian tumors [4], and 60–80% ofthe gastrointestinal tractmors oriasnate from gastrointestinal tra, t (named as Krukenberg tumor) and breast [5, 6]. Depended on CT and magnetic resonance imaging (MRI) findings of ovarian tumors, as it was observed in our case, the masses generally have no distinguished characteristics which contribute to make the judgement about the primary site. Therefore, differentiating between primary ovarian and metastatic adenocarcinoma is crucial since the treatment strategies and prognosis for these diseases are markedly different.

Traditionally, systemic therapy is recommended as the standard therapy for metastatic lung cancer. More remarkably, positive status of driving genes like anaplastic lymphoma kinase (ALK) rearrangement and EGFR mutation in lung cancer with ovarian metastasis has dramatically been reported [7–11] in the latest decade and low toxic targeted therapy would prolong the patients’ lives. For the metastatic lung cancer patients, especially who have received multiple-line treatments, one problem appeared whether it is necessary to perform surgery debulking for accurate diagnosis.

Sufficiently resected tissue from cytoreductive surgery is needed owing to differential diagnosis between primary and metastatic ovarian tumors, as well as an accurate gene detection for afterwards appropriate regimen. Lung cancer is a heterogeneous tumor and many studies have reported the discrepancy of EGFR mutation status between the primary tumor and metastatic lesions from the same patient [12]. Like this case, the previous EGFR status of the primary tumor was totally different with the ovarian metastasis. On the one hand, insufficient tissue quantity or unsatisfied tissue quality might cause the first-time gene result not to present the real gene status. On the other hand, it took long time to possibly develop into various features in different metastatic sites. Whatever, updated driven genes testing of the newly progressive tumor is crucially warranted.

In theory, fewer cancer cells at the start of systemic treatment lead to a higher likelihood of cure, in part due to dose intensity of chemotherapy. Otherwise, it is helpful to make the residual cancer cell clones homogeneous, improving the sensitivity to the afterwards chemotherapies or targeted therapies. Based on studies which have reported the effectiveness of maximal tumor debulking surgery in lengthening the overall survival time compared to the absence of such surgical treatment in Krukenberg tumor [13], the debulking surgery may be a good choice for lung cancer patients with ovarian metastasis.

In conclusion, for the increasing incidence of lung cancer in females and improved survival time, physicians must keep in mind the possibility of ovarian metastasis in clinical course. Debulking surgery followed by optimal systemic treatment, especially targeted therapy may greatly contribute to the prognosis of lung cancer patients with ovarian metastasis. An additional case series will be necessary to confirm our results.

## Data Availability

The datasets used and/or analyzed during the current study available from the corresponding author on reasonable request.

## Abbreviations

CT: computed tomography
EGFR: epidermal growth factor receptor
SD: stable disease
PD: progressive disease
TTF-1: thyroid transcription factor 1
CK: cytokeratin
PAX-8: paired box 8
ER: estrogen receptors
PR: progesterone receptor
MRI: magnetic resonance imaging
ALK: anaplastic lymphoma kinase
